# A modified two-dimensional sensory organization test that assesses both anteroposterior and mediolateral postural control

**DOI:** 10.3389/fresc.2023.1166859

**Published:** 2023-05-22

**Authors:** Andrew R. Wagner, Daniel M. Merfeld

**Affiliations:** ^1^ Department of Otolaryngology—Head and Neck Surgery, The Ohio State University Wexner Medical Center, Columbus, OH, United States; ^2^ School of Health and Rehabilitation Sciences, The Ohio State University, Columbus, OH, United States; ^3^ Department of Biomedical Engineering, The Ohio State University, Columbus, OH, United States; ^4^ Department Speech and Hearing Sciences, The Ohio State University, Columbus, OH, United States

**Keywords:** balance, postural control, sway, vestibular, sensory organization test, posturography, proprioception

## Abstract

**Background:**

The Sensory Organization Test (SOT) was designed to measure changes in postural control in response to unreliable visual and/or proprioceptive feedback. However, secondary to the manipulation of sensory cues in only the sagittal plane, the SOT is capable of only describing postural control in a single direction. The present study aimed to characterize postural responses to a modified SOT designed to concurrently challenge both anteroposterior and mediolateral postural control.

**Methods:**

Twenty-one healthy adult volunteers (30.6 ± 10.2 years) completed the standard anteroposterior one-dimensional (1D) SOT, in addition to a modified SOT with the support surface sway-referenced to both anteroposterior and mediolateral postural sway (two-dimensional, 2D). Our primary analysis concerned a comparison of mediolateral, as well as anteroposterior postural sway measured during the standard one-dimensional (i.e., pitch tilt) and the novel two-dimensional (i.e., roll and pitch tilt) sway-referenced paradigms. Here, postural sway was quantified by calculating the root mean square distance (RMSD) of the center of pressure (CoP) during each trial.

**Results:**

Our data showed that the 2D sway-referenced conditions yielded a selective increase in mediolateral postural sway relative to the standard 1D conditions for both wide (*η*^2^ = 0.66) and narrow (*η*^2^ = 0.78) stance conditions, with anteroposterior postural sway being largely unaffected (*η*^2 ^= 0.001 to 0.103, respectively). The ratio between mediolateral postural sway in the sway-referenced conditions and postural sway in the corresponding stable support surface conditions was greater for the 2D (2.99 to 6.26 times greater) compared to 1D paradigms (1.25 to 1.84 times greater), consistent with a superior degradation of viable proprioceptive feedback in the 2D paradigm.

**Conclusion:**

A modified 2D version of the SOT was shown to provide a greater challenge to mediolateral postural control relative to the standard 1D SOT protocol, putatively as a result of a superior capacity to degrade proprioceptive feedback in the mediolateral direction. Given these positive findings, future studies should investigate the clinical utility of this modified SOT as a means by which to better characterize sensory contributions to postural control in the presence of various sensorimotor pathologies, including vestibular hypofunction.

## Introduction

**Figure 1:**
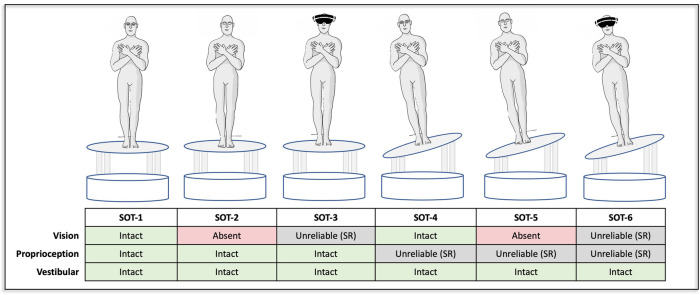
Each of the six conditions of the sensory organization test are shown. All conditions were completed with both a wide and narrow base of support. Per standard protocol the first three conditions (SOT-1, -2, -3) did not include sway-referencing. The final three conditions (SOT-4, -5, -6) were completed using both a one-dimensional (pitch) and a two-dimensional (pitch & roll) sway-reference paradigm. The black masks in SOT-3 and SOT-6 denote the use of VR to provide a sway-referenced visual scene. While VR goggles were worn throughout, they are removed from the graphic in the remaining tasks to allow visualization of the eyes (open vs. closed).

The Sensory Organization Test (SOT) was developed in the 1970's as a way to study how the interactions between vestibular, somatosensory, and visual sensory feedback influence postural control (1–3). The ability of the SOT to identify sensory contributions to balance results from the inclusion of balance tasks designed specifically to manipulate the reliability of visual feedback and/or proprioceptive feedback from the distal lower extremities. This is accomplished through the use of a technique referred to as “sway-referencing” (Figure 1). By moving either the support surface, or visual surround, in phase with an estimate of postural sway, sway-referencing renders the resultant feedback as unreliable. At the distal lower extremities, sway-referencing aims to maintain a near constant angle at the ankle joint and in the visual system it aims to keep a constant distance between the eyes and the visual surround. In both cases, such paradigms place the resultant visual and/or proprioceptive cues in direct conflict with any remaining unperturbed sensory information. As such, the SOT can help to determine (1) an individual's reliance upon a given sensory system (e.g., “visual dependence”) and/or (2) the capacity to remain balanced when forced to primarily use an unperturbed source of sensory feedback (e.g., the vestibular system). Given the ability to parse the reliance upon different sensory modalities, the SOT has become a standard methodology for probing the impact of sensory dysfunction on postural control (4–6).

However, a principal limitation of the standard SOT is its manipulation of sensory feedback in only the anteroposterior direction, which leaves a blind spot in our understanding of mediolateral balance control. The platform and/or visual scene are sway-referenced relative only to an estimate of pitch plane postural sway, and as such, only the sensory cues relevant to the control of balance in the pitch (i.e., anteroposterior) direction are made to be unreliable. This is reflected by the standard summary output of the SOT, the Equilibrium Score, which describes the maximal displacement of the center of gravity in only the anteroposterior direction (7). This limitation bears relevance to the testing of clinical populations secondary to, (1) data showing that mediolateral or “roll plane” postural control is an important predictor of fall risk (8), and of fall related injury (9–11) and (2) the fundamental knowledge that the postural control system is inherently multidimensional, as humans must simultaneously control the orientation of their body in both the roll and pitch directions during daily life.

Therefore, the purpose of the present study was to test a modified two-dimensional (2D) SOT paradigm designed to manipulate the fidelity of proprioceptive cues in both the roll and pitch directions. In addition, we aimed to determine if the width of the base of support influenced postural sway during both the standard 1D, as well as novel 2D sway-referenced conditions. We hypothesized that the 2D sway-referenced conditions would yield an increase in ML postural sway compared to the 1D conditions, and that AP postural sway would remain unchanged.

## Methods

### Study design

Participants were recruited from The Ohio State University and The Ohio State University Wexner Medical Center. Exclusionary criteria included a history of vestibular disorders, alternative neurological disease or injury, uncorrected visual impairment, or recent (within 6 months) orthopedic injuries/surgeries. All individuals provided informed consent and the study was approved by the Ohio State University Institutional Review Board. Testing occurred in a single session that lasted no longer than 60 min (including rest). The order of testing was randomized and counterbalanced allowing an equal proportion of individuals to start with each combination of sway-referencing (1D vs. 2D) and stance width (wide vs. narrow) (Table 1).

**Table 1:** Order of sensory organization test conditions.

	*N* = 5		*N* = 5		*N* = 6^a^		*N* = 5	
	Sway-ref	Width	Sway-ref	Width	Sway-ref	Width	Sway-ref	Width
Block 1	1D	Wide	2D	Wide	1D	Narrow	2D	Narrow
Block 2	2D	Wide	1D	Wide	2D	Narrow	1D	Narrow
Block 3	1D	Narrow	2D	Narrow	1D	Wide	2D	Wide
Block 4	2D	Narrow	1D	Narrow	2D	Wide	1D	Wide

Wide = stance with the heads of the fifth metatarsals 33 cm apart. Narrow = stance with the heads of the first metatarsals 1.5 cm apart, 1D = sway-referencing only in the pitch plane, 2D = sway-referencing both in the pitch and roll planes.

^a^ Six subjects completed the third test order as we over-recruited to 21 to account for dropouts or data collection errors.

### Equipment and procedures

**Figure 2:**
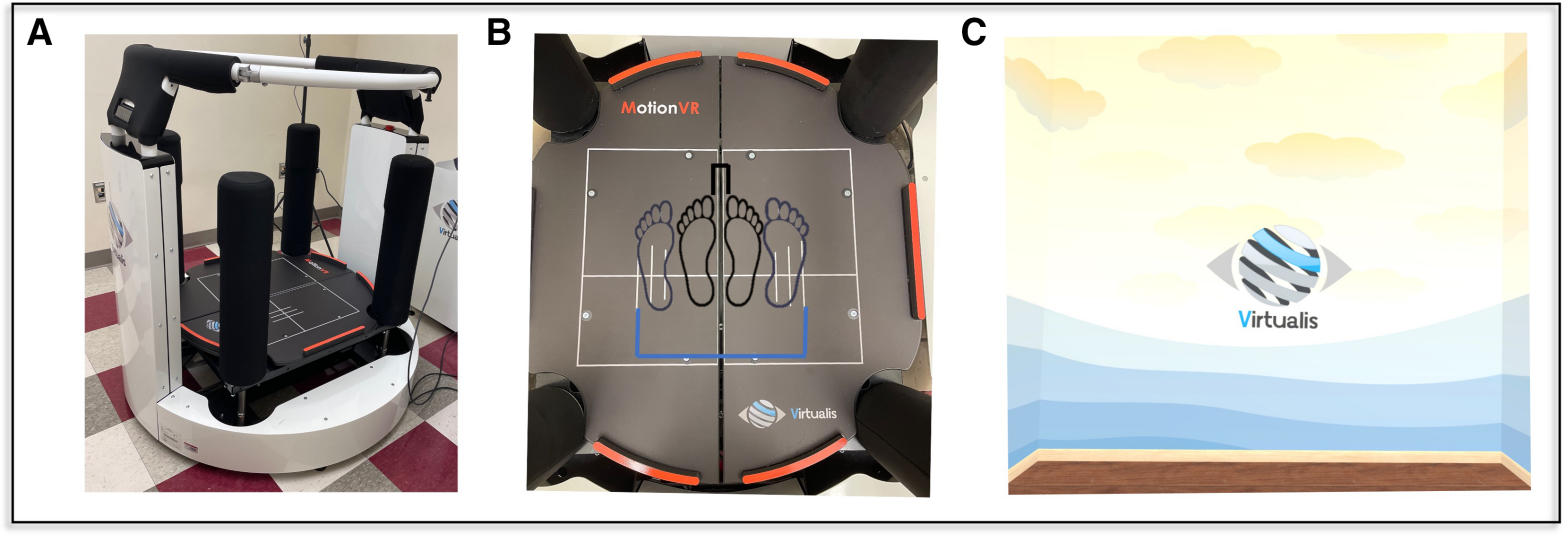
The virtualis motionVR (**A**) platform was used to perform the SOT test protocol. Embedded force plates (**B**) recorded the CoP while the participant stood with a narrow (black) and wide (blue) base of support. A virtual room (**C**) was used as visual feedback; in visual sway-referenced conditions the image moved in concert with the head (SOT3, SOT6), in normal vision conditions (SOT1, SOT4) the virtual room appeared to remain stationary relative to the participant's sway. For conditions without visual cues (SOT2, SOT5) the headset went black, removing all visual cues, and subjects were asked to close their eyes.

Each SOT task was performed using a Virtualis (Perrault, France) MotionVR platform that can provide simultaneous sway-referencing in both the mediolateral and anteroposterior directions. The Virtualis system consists of a motion platform, controlled via four linear actuators that yield a rotation axis 29 cm below the platform surface, synchronized with an HTC Vive Virtual Reality headset through Steam VR (v2.0) (Figure 2A). During each balance trial, two tri-axial force plates rigidly contained within the moving platform sampled center of pressure data at rate of 90 Hz. During “sway-referenced” trials, the platform was tilted in concert with an estimate of the displacement of the center of gravity, consistent with traditional SOT testing. The HTC Vive VR headset was used to provide both a stationary, as well as a sway-referenced visual scene [per eye resolution of 1,080 × 1,200 pixels and a 108-degree field of view (12)] (Figure 2C). Motion of the visual scene and platform was produced with a resolution of 0.011 s (90 Hz).

Instructions were provided before each task to inform the participant of the visual environment (“eyes open” or “eyes closed”) and platform condition (“the platform will be stationary” or “the platform may move”). The participant was also instructed to minimize volitional movement and to simply remain upright and as still as possible. To avoid unintended tactile cues, a harness was not worn, but instead a ring around the platform was used to provide assistance in the event of a fall; a trained operator was also present and available immediately to assist if a loss of balance occurred. Each of the SOT tasks lasted 20 s and were repeated three times. Between tasks (i.e., after 1 min of testing), the participant was asked to step-down from the platform to rest and to allow for zeroing of the force plates.

### Test conditions

Each participant completed a total of 18 unique SOT tasks, with each task consisting of three trials of 20 s each. The first three SOT conditions (each with a fixed support surface) were performed with a narrow (first metatarsals 1.5 cm apart), as well as wide stance (fifth metatarsals 33 cm apart), to allow comparisons to the sway-referenced trials (Figure 1). The latter three conditions of the SOT that include a sway-referenced support surface — SOT-4, SOT-5, and SOT-6 — were completed (a) with the platform sway-referenced in only the pitch plane [i.e., standard one-dimensional (1D) sway-referencing], or (b) with the platform sway-referenced to both pitch and roll postural sway [i.e., two-dimensional (2D) sway-referencing] (Figure 1). Each of the 1D and 2D sway-referenced conditions were also completed using both a wide and narrow base of support (Figure 2B). The base of support for the wide stance trials was consistent with the recommended stance width for individuals between 65 and 78 inches in the standard SOT assessment (13). A fixed width, rather than a width dictated by height, was used to standardize the comparison to the narrow stance trials. Prior to each condition, we confirmed the alignment of the feet using markers located on the platform and also confirmed that the malleoli were aligned with the axis of rotation in the anteroposterior direction.

**Figure 3:**
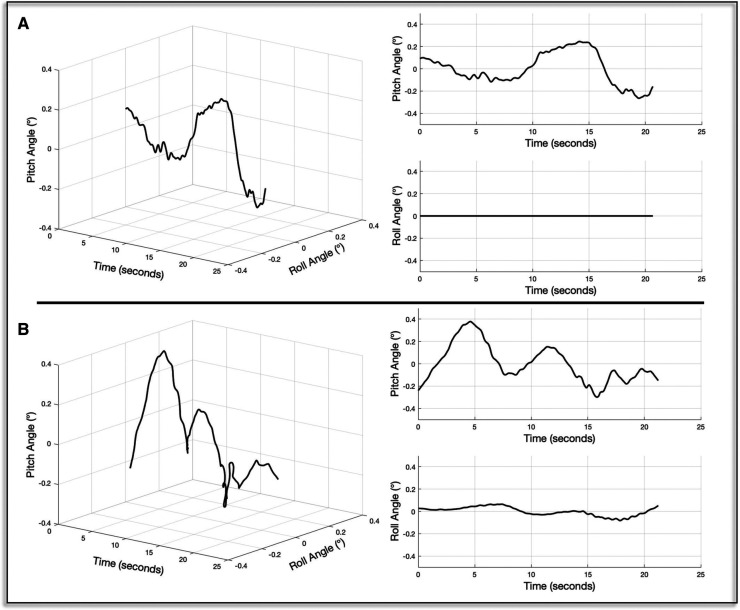
The time series of platform tilts for exemplar one- (**A**) and two-dimensional (**B**) sway-reference trials are shown. To the right, the pitch and roll components of each motion stimulus are shown.

Sway-referencing has been described previously at length (14), so here we provide only a terse overview to highlight the differences between the 1D and 2D conditions. In the traditional “one-dimensional” (1D) SOT, during each of the sway-referenced conditions the platform is tilted in synchrony with an estimate of the body's center of gravity in the pitch plane — i.e., forward body sway causes the front of the platform to pitch downward and backward sway causes the front of the platform to pitch upward (Figure 3A). This motion serves the primary purpose of minimizing the typical change in sagittal plane ankle motion experienced during sway with a fixed base of support. In the present study we compared this protocol to a “two-dimensional (2D)” sway-referenced condition whereby the platform instead tilted in response to estimates of sway angle in both the pitch and roll planes (Figure 3B) — e.g., diagonal sway forward and to the right yielded a simultaneous forward pitch of the platform alongside a rightward tilt in the roll plane.

### Analysis of CoP data

CoP data were recorded during each trial and analyzed off-line to calculate the outcome of interest. The CoP data were first low-pass filtered using a 4th order zero-phase-lag butterworth filter with a cutoff of 25 Hz (*filtfilt.m*; MATLAB, Natick, MA). The root mean square distance (RMSD) was then calculated by taking the standard deviation of the zero-meaned and filtered CoP signal (15–17). RMSD values were calculated separately for the ML and AP CoP. Secondary to potential learning effects or transient responses, the median values are reported from each block of three SOT trials. The standard outcome for the sensory organization test, the Equilibrium Score (7), was not calculated as such values only consider AP postural sway. In addition, the Equilibrium Score is based upon an assumed limit of stability (12.5°), which applies only to wide stance, 1D (i.e., pitch plane) sway-referenced conditions.

In addition to the raw sway responses, we also calculated normalized sway ratios by taking the median RMSD values in SOT-4, SOT-5, and SOT-6 and dividing them by the RMSD values captured in the corresponding SOT conditions that were identical, with the exception of providing a stable base of support (i.e., SOT-1, SOT-2, and SOT-3 respectively) [Figure 1, Equations (1)–(3)]. As the sway-referencing paradigm is intended to degrade the fidelity of proprioceptive inputs from the distal lower extremities, the normalized sway ratios were calculated as means to quantify the success of each paradigm (i.e., greater sway ratio = greater decrement in balance performance with the manipulation of proprioceptive cues via sway-referencing).


(Eq. 1) $$Norm_{SOT4}=\;\frac{RMSD_{SOT4}}{RMSD_{SOT1}}$$

(Eq. 2) $$Norm_{SOT5}=\;\frac{RMSD_{SOT5}}{RMSD_{SOT2}}$$

(Eq. 3) $$Norm_{SOT6}=\;\frac{RMSD_{SOT6}}{RMSD_{SOT3}}$$

### Data analysis

A 2 × 3 repeated measures analysis of variance (RM-ANOVA) model was used to determine the differences in postural sway between the 1D and 2D sway-referenced conditions for each of the three sway-referenced conditions of the SOT (SOT-4, -5, and -6). Four separate models were run to separately analyze AP, as well as ML postural sway, in both the narrow and wide stance conditions. In each model, a sway-reference (1D vs. 2D) times SOT condition (SOT-4, SOT-5, SOT-6) interaction term was also tested. After each of the four models, we performed pairwise comparisons to determine the differences in postural sway between the 1D and 2D sway-referenced conditions (3 comparisons each × 4 models = 12 comparisons total); the reported *p*-values were corrected using the Bonferroni method (*p*-value × 12). Although the CoP data were found to deviate from normality (as tested by visualization of normal probability plots and by the results of Shapiro-Wilk test), ANOVA models have been shown to be robust to such violations (18). Normalized sway ratios for the 1D and 2D trials were compared using pairwise Wilcoxon Rank-Sum tests of medians due to the rightward skew of the distributions. The six comparisons for wide stance and six comparisons for narrow stance were corrected using the Bonferroni method (*p*-value × 12). We also calculated intraclass correlation coefficients (ICC) and the corresponding 95% confidence intervals for each of the different sway-referenced SOT conditions. The ICC model (a) included both random and fixed effects, (b) was based upon a single measure at each of three time points (Trial 1, 2, and 3) and (c) yielded a measure of absolute agreement (including both random and systemic variance) (Stata v.17, College Station, TX). Here we defined repeatability as low (ICC < 0.5), moderate (ICC = 0.5 to 0.75), good (ICC = 0.75 to 0.9), or excellent (ICC > 0.9) (19).

## Results

### Differences in postural sway between the 1D and 2D sway-referenced conditions

#### Mediolateral sway

**Figure 4:**
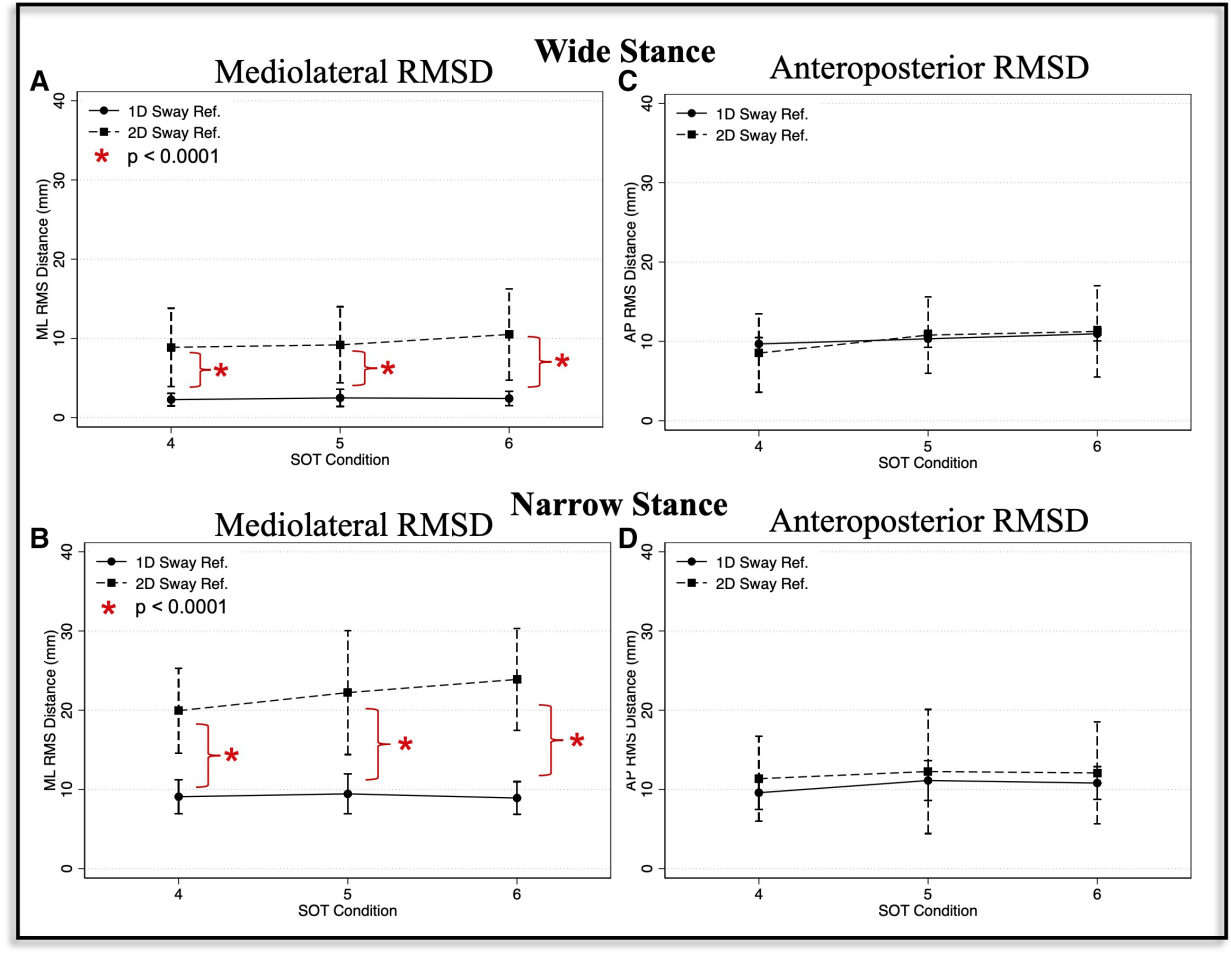
Mediolateral (**A, B**) and anteroposterior (**C, D**) root mean square distance (RMSD) values are shown for the one-dimensional (1D, circle with solid line) and two-dimensional (2D, square with broken line) sway-referenced trials, and for both wide (**A, C**) and narrow (**B, D**) stance conditions. Red asterisks indicate significant differences (*p* < 0.0001) between the 1D and 2D conditions based on pairwise comparisons.

In the analysis of SOT trials that used a wide base of support, we identified a significant main effect of sway-reference condition (1D vs. 2D) (*η*^2^ = 0.66, F(1,100) = 189.90, *p* < 0.0001) on the RMSD of the ML CoP. The effect of 2D vs. 1D sway-referencing was not significantly modified by SOT condition (sway-reference times SOT condition interaction; *η*^2 ^= 0.017, F(2,100) = 0.86, *p* = 0.43). Post-hoc pairwise comparisons showed that the ML RMSD was significantly increased for the 2D compared to 1D-sway-reference trials in SOT-4 [Diff = 6.6, *p* < 0.0001, 95% CI (3.98, 9.23)], SOT-5 [Diff = 6.70, *p* < 0.0001, 95% CI (4.07, 9.32)], and SOT-6 [Diff = 8.08, *p* < 0.0001, 95% CI (5.46, 10.71)] (Figure 4A, Table 2).

**Table 2:** Comparison of postural sway between the one-dimensional (1D) and two-dimensional (2D) sway-referenced conditions.

1D and 2D RMSD values						
		1D sway-ref.	2D sway-ref.	*t*	*p*-value	95% CI
Wide						
AP	SOT-4	9.68 ± 4.29	8.52 ± 3.06	−1.59	>0.99	−3.28, 0.97
	SOT-5	10.33 ± 3.46	10.80 ± 4.30	0.65	>0.99	−1.66, 2.6
	SOT-6	10.96 ± 4.04	11.25 ± 4.21	0.40	>0.99	−1.84, 2.42
ML	SOT-4	2.27 ± 0.8	8.87 ± 4.94	7.37	<0.0001	3.98, 9.23
	SOT-5	2.49 ± 1.08	9.18 ± 4.81	7.48	<0.0001	4.07, 9.32
	SOT-6	2.41 ± 0.91	10.50 ± 5.76	9.02	<0.0001	5.46, 10.71
Narrow						
AP	SOT-4	9.59 ± 3.69	11.35 ± 3.99	2.49	0.18	−0.32, 3.86
	SOT-5	11.13 ± 3.75	12.26 ± 4.61	1.59	>0.99	−0.95, 3.22
	SOT-6	10.81 ± 4.08	12.08 ± 3.07	1.80	0.91	−0.8, 3.36
ML	SOT-4	9.08 ± 2.13	19.94 ± 5.35	9.25	<0.0001	7.42, 14.3
	SOT-5	9.44 ± 2.52	22.22 ± 7.83	10.88	<0.0001	9.34, 16.22
	SOT-6	8.92 ± 2.07	23.88 ± 6.44	12.74	<0.0001	11.53, 18.41

Comparisons made were the result of pairwise comparisons performed following the repeated measures ANOVA. Reported *p*-values and confidence intervals are corrected using the Bonferroni method (12 comparisons).

AP, anteroposterior; ML, mediolateral; SOT, sensory organization test.

For narrow stance trials, the effect of 1D vs. 2D sway-referencing was also significant, and the size of the effect was larger than for the wide stance trials (*η*^2^ = 0.78, F(1,100) = 360.24, *p* < 0.0001). We also identified a borderline significant interaction between SOT condition and sway-reference paradigm (1D vs. 2D) (*η*^2 ^= 0.058, F(2,100) = 3.06, *p* = 0.051). Post-hoc pairwise comparisons showed that the ML RMSD was significantly increased for 2D vs. 1D sway-referencing for SOT-4 [Diff = 10.86, *p* < 0.0001, 95% CI (7.42, 14.3)], SOT-5 [Diff = 12.78, *p* < 0.0001, 95% CI (9.34, 16.22)] and SOT-6 [Diff = 14.97, *p* < 0.0001, 95% CI (11.53, 18.41)] (Figure 4B, Table 2).

#### Anteroposterior sway

Sway-reference condition (1D vs. 2D) did not show a significant main effect on the RMSD of the AP CoP in the wide stance trials (*η*^2 ^= 0.00099, F(1,100) = 0.1, *p* = 0.75). The effect of 2D vs. 1D sway-referencing on AP postural sway was also not significantly modified by SOT condition (*η*^2 ^= 0.029, F(2, 100) = 1.50, *p* = 0.23). Pairwise comparisons showed that the RMSD of the AP CoP was not significantly different between the 1D compared to 2D sway-referenced conditions for SOT-4 [Diff = − 1.16, *p* > 0.99, 95% CI (−3.28, 0.97)], SOT-5 [Diff = 0.47, *p* > 0.99, 95% CI (−1.66, 2.6)], or SOT-6 [Diff = 0.29, *p* > 0.99, 95% CI (−1.84, 2.42)] (Figure 4C, Table 2).

We did however identify a significant, albeit small (*η*^2 ^= 0.103), main effect of 2D vs. 1D sway-referencing on the RMSD of the AP CoP in the narrow stance trials [F(1,100) = 11.50, *p* = 0.001]. However, the effect of 1D vs. 2D sway-referencing was not significantly influenced by SOT condition (*η*^2 ^= 0.0044, F(2, 100) = 0.22, *p* = 0.804) and pairwise comparisons showed that the AP RMSD was not significantly different between the 1D and 2D trials for any of the individual SOT conditions [SOT-4: Diff = 1.77, *p* = 0.18, 95% CI (−0.316, 3.86); SOT-5: Diff = 1.13, *p* > 0.99, 95% CI (−0.95, 3.22); SOT-6: Diff = 1.28, *p* = 0.91, 95% CI (−0.81, 3.36)] (Figure 4D, Table 2).

#### Normalized sway ratios

**Figure 5:**
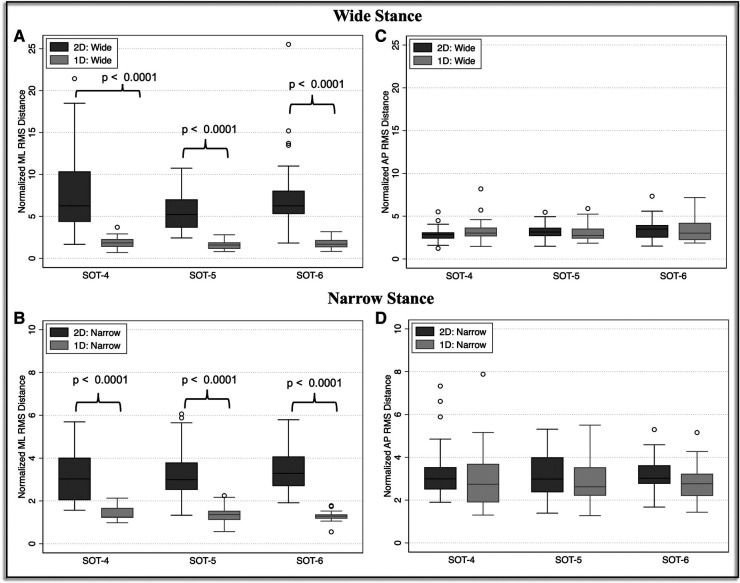
The median and interquartile range (IQR) is shown for each of the mediolateral (**A, B**) and anteroposterior (**C, D**) normalized sway ratios. Ratios were calculated by dividing the RMSD in the sway-referenced conditions by the RMSD in the corresponding condition that included a stable support surface: SOT-4/SOT-1, SOT-5/SOT-2, and SOT-6/SOT-3. In each plot, the ratios calculated from the 2D trials (dark grey) are shown against the ratios calculated from the 1D trials (light grey). *P*-values reflects the results of a Wilcoxon Rank Sum test of medians between the 1D and 2D sway-reference conditions. *P*-values are Bonferroni corrected (corrected according to 6 comparisons for wide stance and 6 comparisons for narrow stance).

The ratios describing the RMSD in the sway-referenced conditions relative to the RMSD in the corresponding stable support conditions — (a) SOT-4/SOT-1, (b) SOT-5/SOT-2, and (c) SOT-6/SOT-3—were also compared between trials that used a 2D compared to a 1D sway-referencing protocol. For ML postural sway, the normalized sway ratios were significantly increased for the 2D relative to the 1D sway-referenced conditions, suggesting a greater degradation of ML proprioceptive cues in the 2D condition (Figures 5A,B). In the wide stance trials, ML RMSD values in the 1D sway-referenced conditions were increased 1.59 to 1.84 times relative to sway in the stable support surface conditions (i.e., sway ratios between 1.59 and 1.84 for SOT-4, -5, and -6). By contrast, ML RMSD values in the 2D sway-referenced conditions were between 5.22 to 6.26 times higher than in the stable support surface conditions (Table 3). Similarly, in narrow stance mediolateral sway ratios for the 1D sway-referenced trials were between 1.25 to 1.36 compared to 2.99 to 3.29 for the 2D trials (Table 3). For each of these comparisons, the difference in sway ratios between 1D and 2D conditions was significant at *p* < 0.001.

**Table 3:** Median ratios between SOT-4/SOT-1, SOT-5/SOT-2 and SOT-6/SOT-3 are reported along with the IQR.

Normalized sway ratios					
		1D ratio	2D ratio	Difference	*p*-value
Wide					
AP	SOT-4	3.03 (2.64–3.66)	2.85 (2.39–3.10)	−0.43	0.51
	SOT-5	2.75 (2.38–3.54)	3.15 (2.68–3.65)	0.032	>0.99
	SOT-6	3.02 (2.23–4.22)	3.49 (2.50–3.96)	0.008	>0.99
ML	SOT-4	1.84 (1.38–2.30)	6.26 (4.36–10.36)	5.76	<0.001
	SOT-5	1.59 (1.13–1.91)	5.22 (3.66–7.03)	4.18	<0.001
	SOT-6	1.69 (1.32–2.18)	6.26 (5.30–8.06)	6.21	<0.001
Narrow					
AP	SOT-4	2.74 (1.89–3.69)	2.99 (2.5–3.54)	0.46	0.14
	SOT-5	2.60 (2.21–3.53)	2.98 (2.37–4.00)	0.20	>0.99
	SOT-6	2.77 (2.19–2.32)	3.02 (2.76–3.63)	0.41	0.55
ML	SOT-4	1.25 (1.21–1*.*67)	3.03 (2.04–4.02)	1.78	<0.001
	SOT-5	1.36 (1.11–1.54)	2.99 (2.52–3.79)	1.92	<0.001
	SOT-6	1.29 (1.17–1.37)	3.29 (2.70–4.08)	2.18	<0.001

The difference reflects the average difference between the 1D and 2D ratios. *P*-values reflects the results of a Wilcoxon rank sum test of medians between the 1D and 2D sway-reference conditions. *P*-values are Bonferroni corrected (corrected according to 12 comparisons).

Regarding AP postural sway, the normalized sway ratios did not significantly differ between any of the 1D and 2D sway-referenced trials, consistent with the 1D and 2D sway-referencing paradigms yielding similar increases in the AP RMSD relative to the stable support surface conditions (Figure 5C, Table 3). This finding was true both for wide (1D Ratios = 2.75 to 3.03, 2D ratios = 2.85 to 3.49), as well as narrow stance (1D Ratios = 2.6 to 2.77, 2D Ratios = 2.98 to 3.02) conditions (Figure 5D, Table 3).

#### Repeatability of the 2D SOT conditions

Table 4 shows the ICC values for SOT-4, SOT-5, and SOT-6 during both the 1D and 2D sway-referenced conditions. Overall, the 2D sway-referencing paradigm yielded moderate to good agreement between trials for the ML RMSD values captured during both the narrow and wide stance trials (Table 4, Range: 0.566 < ICC < 0.763**)**. With a single exception (SOT-5 in wide stance) where the 1D condition yielded an 11% higher ICC value, the ICC values were higher for the 2D compared to 1D trials. For sway measured in the AP direction, we observed a similar but slightly lower degree of reliability (Table 4, Range: 0.411 < ICC < 0.669), with all but 2 of the conditions (SOT-4, wide and SOT-5, narrow) showing greater agreement for the 2D compared to 1D trials.

**Table 4:** Intraclass correlation coefficients (ICC) with a 95% confidence intervals are shown for each of the sway-referenced test conditions.

	Wide stance		Narrow stance	
	1D sway-referenced	2D sway-referenced	1D sway-referenced	2D sway-referenced
ML RMSD				
SOT 4	0.496 (0.233, 0.728)	0.763 (0.581, 0.887)	0.544 (0.288,0.759)	0.566 (0.314,0.773)
SOT 5	0.66 (0.436,0.830)	0.587 (0.341,0.787)	0.340 (0.072,0.617)	0.697 (0.485,0.851)
SOT 6	0.565 (0.313,0.773)	0.588 (0.341,0.787)	0.558 (0.305 to 0.769)	0.745 (0.553,0.877)
AP RMSD				
SOT 4	0.568 (0.316,0.774)	0.524 (0.265,0.747)	0.464 (0.198,0.707)	0.585 (0.338,0.785)
SOT 5	0.467 (0.201,0.709)	0.669 (0.447,0.835)	0.556 (0.302,0.767)	0.411 (0.142,0.669)
SOT 6	0.374 (0.104,0.642)	0.553 (0.299,0.765)	0.454 (0.187,0.700)	0.515 (0.255,0.741)

The ICC formula used a mixed effect model based upon a single measure and provides a measure of absolute agreement (including both random and systematic variance). Stata v.17 (College Station, TX).

## Discussion

Our primary hypothesis was that the 2D sway-referencing paradigm, owing to the manipulation of roll plane proprioceptive feedback, would yield an increase in mediolateral postural sway when compared to the standard 1D SOT protocol. Consistent with this hypothesis, our data showed that the RMSD of the ML CoP was significantly increased for the 2D sway-referenced trials in both narrow and wide stance conditions. We also showed that the 2D and 1D trials yielded similar amounts of postural sway in the AP direction, indicative of a similar level of challenge to AP postural control. In our secondary analysis, we showed that when the RMSD values for the sway-referenced conditions were compared to postural sway in the corresponding fixed support surface conditions (i.e., without sway-referencing), the 2D protocol yielded significantly greater increases in ML sway compared to the 1D protocol, consistent with a greater degradation in proprioceptive feedback in the 2D paradigm. Below we discuss the implications of these findings in the context of the available literature, as well as putative applications for using the modified 2D SOT to better characterize human postural control in both health and disease.

### Influences of a 1D vs. a 2D sway-referenced surface on postural control

To our knowledge, this is the first study to characterize postural responses to a two-dimensional (i.e., in both roll and pitch planes) sway-referenced support surface. Allum and colleagues did however separately measure postural sway in response to a 1D roll, as well as a 1D pitch sway-referenced support surface. When compared to a standard “foam standing” condition, they found ML postural responses to be reduced for the 1D pitch sway-referenced condition (20). Foam standing, although methodologically distinct from a sway-referenced support surface, is conceptually similar to our 2D sway-referencing paradigm, as each degrades the efficacy of ankle proprioceptive feedback in both the AP and ML planes. Consistent with their finding, here we showed less ML postural sway in the 1D (pitch only) compared to the 2D (pitch and roll) sway-referenced conditions.

We posit that the ability for individuals to minimize mediolateral sway in a 1D sway-referenced condition likely results from the persistent availability of reliable proprioceptive cues derived from the stationary (in the roll plane) support surface. During the 1D sway-referenced condition, the platform fails to tilt in the roll plane, and thus, any off-axis mediolateral sway yields stimulation of distal receptors in the lower limbs, providing accurate information about the orientation of the body relative to support surface. Our data show that when this feedback is made to be unreliable through use of a 2D sway-referenced condition — whereby mediolateral sway is met with a corresponding roll tilt of the surface — that the control of postural sway in the roll plane is impaired, yielding an increase in the mediolateral RMSD of the CoP. This capacity for the 2D condition to further degrade proprioceptive inputs represents the primary advantage of this protocol over the 1D SOT. However, the ability of the different sway-referencing paradigms to manipulate proprioceptive cues can better be appreciated by looking at the ratio between (a) the amount of postural sway in conditions with altered proprioceptive cues (sway-referenced support) relative to (b) the amount of postural sway in conditions with intact proprioceptive cues (fixed support surface).

The SOT is designed such that the final three conditions (SOT-4, SOT-5, and SOT-6) mirror the first three conditions, with the exception that SOT-4 through -6 include a sway-referenced support surface (i.e., identical vestibular and visual cues). Thus, by calculating ratios between postural sway in SOT-4 and SOT-1, SOT-5 and SOT-2, and SOT-6 and SOT-3 we can determine to what extent the removal of viable proprioceptive inputs—by way of each of the different sway-referencing paradigms—influences postural control. As both the visual and vestibular feedback are fixed for each comparison (i.e., eyes closed, open, or with vision sway-referenced), increases in sway relative to quiet stance can therefore be attributed to a greater deterioration of proprioceptive feedback. When analyzing mediolateral postural sway, each of the six unique ratios (i.e., SOT-4/1, SOT-5/2, SOT-6/3 for both wide and narrow stance) were significantly greater for the 2D relative 1D sway-referencing paradigm, consistent with the hypothesis that the 2D paradigm more successfully limits the use of proprioceptive cues from the support surface.

In the analysis of AP postural sway, ratios calculated from the 1D and 2D sway-referenced trials were instead similar, suggesting a similar manipulation of pitch plane support surface cues. The similarities in AP postural sway between the 1D and 2D sway-referenced conditions lends further support to our hypothesis that the 2D sway-referenced condition diminishes proprioceptive feedback in two-dimensions, rather than causing a compensatory strategy that favors body sway in the ML, as opposed to the AP, direction. The ability to more completely alter proprioceptive inputs during stance holds potential promise for the development of improved methods for evaluating patients with presumed sensorimotor impairments, including vestibular dysfunction.

### Implications for testing clinical populations

The SOT has become one of the gold standard methods for characterizing the sensory contributions to balance performance. Principle amongst its clinical uses is in the evaluation of the dizzy patient (21). When considered alongside laboratory and oto-neurological findings, greater postural sway in the presence of unreliable visual and proprioceptive feedback (i.e., SOT-5) has been used to help identify a lesion to the vestibular periphery. However, in isolation, the traditional 1D SOT lacks sufficient sensitivity and specificity to serve as a suitable tool for diagnosing a peripheral vestibular lesion as the potential cause of balance dysfunction (22–24). One potential explanation for this limitation is the inability to sufficiently manipulate the veracity of extra-vestibular sensory feedback in the sway-referenced conditions, resulting in the continued reliance upon proprioceptive inputs.

Here, in a cohort of healthy adults without vestibular pathology, we showed that mediolateral postural sway was only slightly increased in the standard 1D sway-referenced conditions relative to quiet stance (i.e., normalized sway ratios of 1.25 to 1.84). By comparison, in the novel 2D sway-referenced conditions, the increase in ML postural sway was striking when compared to quiet standing (i.e., normalized sway ratios of 2.99 to 6.26). We posit that the greater availability of mediolateral support surface cues in the traditional 1D SOT may potentially mask the impact of a vestibular lesion. A compensatory prioritization of proprioceptive cues in the roll plane would explain the mitigation in AP sway (i.e., as quantified by the equilibrium score) seen in a subset of patients with compensated vestibular lesions. While speculative, it is also reasonable to conjecture that the previously reported learning effect of the standard SOT could also be a manifestation of a learned behavior to rely more upon the reliable mediolateral support surface cues (25). The proposed 2D SOT should be tested in individuals with well-characterized vestibular lesions to determine if the greater ability to degrade proprioceptive cues may aide in the differentiation between vestibular mediated balance deficits and alternative causes of postural instability.

### Benefits of measuring sensory contributions to mediolateral postural control

In addition to minimizing the contributions of proprioceptive inputs, the 2D sway-referenced paradigm also provides an opportunity to characterize sensory contributions to ML postural control. Previous data suggests that an increase in ML, as opposed to AP, postural sway represents a strong predictor of future falls. Maki, et al. 1994 showed that the RMSD of the ML CoP measured during an eyes closed, quiet stance balance task was the single best predictor of falls in the 12-month period that followed the assessment (80% sensitivity, 46% specificity) (8). In addition, mediolateral postural control may be particularly relevant to the avoidance of serious fall related injuries, including hip fracture (9–11, 26). Greenspan showed that older adults who experienced a fall related hip fracture were more than five times as likely to have experienced a fall in the lateral direction (Odds Ratio = 5.7, 95% CI = 1.7, 18) (11). Nevitt and colleagues similarly found that falls in the lateral direction were a strong predictor of fall related hip fracture (Odds Ratio = 3.3, 95% CI = 2.0, 5.6) (27). The association between lateral instability and hip fracture appears to result from the mechanical stress caused by direct contact between the lateral hip and the ground, as Hayes and colleagues showed that falling directly on the lateral hip was associated with more than a 21-times increase in the odds of experiencing a fall-related hip fracture (Odds Ratio = 21.7, 95% CI = 8.2, 58) (26). Since 98% of hip fractures among the elderly are fall related (28), and lateral instability is predictive of hip fracture, we posit that an improved understanding of mediolateral postural control is critical to the eventual development of improved methods for preventing fall related morbidity and mortality. Whereas the traditional SOT minimizes challenge to mediolateral postural secondary to (a) the wide base of support and (b) the manipulation of only sagittal plane sensory feedback, the novel 2D SOT paradigm — in particular when paired with a narrow base of support — is well suited to aide in such efforts by helping to characterize the relative effects of sensory dysfunction on mediolateral postural control.

It is also worth mentioning that in addition to the specific assessment of ML postural control, the 2D sway-referenced protocol also measures postural responses generated simultaneously in both the AP and ML planes. As humans negotiate their environments, rarely, if ever, is balance perturbed in only a single plane of motion. Even in the atypical event of an isolated stimulus (i.e., a trolley starts suddenly from a stop), such stimuli are rarely aligned perfectly with a single plane of the human body, and therefore require a complex, multi-dimensional motor response. Fittingly, the neuromuscular response to a balance perturbation has been shown to consistent of synergistic responses generated through a combination of muscles acting in both the sagittal and coronal planes (29, 30). As a result, assessments of mediolateral, as well as anteroposterior, postural control in the context of a 2D task may better represent the integrity of the sensorimotor system. Future studies should test this speculation by determining the capacity for a 2D, as compared to standard 1D, sway-reference paradigm to predict fall risk, and/or fall related injury.

### Use of a narrow vs. wide base of support

Aside from the sway-referencing of the support surface, an alternative consideration for the challenge of mediolateral postural control is the width of the base of support. The traditional SOT manipulates stance width based upon subject height, using one of three standardized widths. For each height, the width selected yields a comfortable base of support. Here we aimed to determine how stance width influenced the relationship between 1D and 2D sway-referenced conditions. We found a stronger effect of sway-referencing (1D vs. 2D) on ML postural sway for the narrow stance (*η*^2^ = 0.78) compared to wide (*η*^2^ = 0.66) stance trials, consistent with a larger overall increase in ML postural sway for the 2D compared to 1D trials when in a narrow stance posture. These findings support that narrow stance may therefore be the preferred method by which to challenge ML postural control in the 2D sway-referenced condition.

Yet, we found that the normalized sway ratios (describing postural sway in SOT-4, -5, and-6 relative to the unperturbed quiet standing conditions) were greater for the wide compared to narrow stance trials. In wide stance, the use of a 2D sway-referenced surface increased postural sway by a factor of 5.22 to 6.26, whereas for narrow stance the ratios were only between 2.99 and 3.29. While both wide and narrow stance conditions showed a dramatic increase in sway relative to quiet standing, the difference between the two is worth noting. This difference is likely a result of the very small amounts of ML postural sway recorded in the wide (1.36 to 1.76 mm) compared to narrow (6.67 to 7.33 mm) quiet standing conditions, as the ML RMSD values for narrow stance in SOT-4 through SOT-6 (19.94 to 23.8 mm) were approximately double those measured in the wide stance trials (8.87 to 10.50 mm).

The choice of a narrow vs. wide base of support when implementing the 2D version of the SOT may therefore depend upon the goal of the study. If the goal is to test how postural control in the sway-referenced support surface conditions (SOT4-6) differ from the stable support surface conditions (SOT1-3), then the use of a wider base of support may be preferred. However, if the intent of the assessment is to probe 2D postural control, then narrow stance posture should be chosen due to the heightened challenge to mediolateral postural control when standing with a narrow base of support. Based upon our data, we posit that the narrow stance posture provides a suitable compromise, whereby (a) postural sway in the AP and ML directions is clearly distinct for the 2D sway-referenced tasks relative to the stable support surface conditions and (b) ML postural sway is sufficiently challenged, without compromise to the concurrent assessment of AP postural sway. Nevertheless, such claims should be tested in individuals with a broader range of functional capacities, as this may reveal unique insights, as well as provide valuable data into the feasibility of completing this narrow 2D protocol in individuals with more severe balance impairment. We do highlight that in a yet to be peer-reviewed thesis study (31), 19 out of 21 subjects over the age of 65 were able to complete all six conditions of the described 2D “narrow stance” SOT protocol.

### Limitations

The study was completed in a sample of young, healthy adults, and as such the findings cannot be assumed to represent the behavior of individuals with balance dysfunction. These data instead provide the expected physiologic response to this novel test paradigm, from which future studies should compare the responses of individuals with various types of sensorimotor impairment (e.g., vestibular hypofunction, peripheral neuropathy). We also utilized a VR based SOT, which differs from the traditional SOT paradigm that utilizes a mechanical visual scene. As this test condition was used for both the 1D and 2D SOT tasks, such differences are unlikely to have influenced our results on a within subject basis. Finally, we did not include the standard output of the SOT, the Equilibrium Score, as this metric is not conducive to the novel SOT conditions used here. Specifically, no standard for “maximal” sway angle has been developed for ML postural sway or for narrow stance conditions. We did however opt to utilize a measure of sway displacement (RMSD), as this captures a similar construct as the displacement-based Equilibrium Score.

## Conclusions

We showed that a two-dimensional sway-referenced SOT protocol, whereby proprioceptive cues were manipulated in both the pitch and roll planes, yielded an increase in mediolateral postural sway when compared to the standard one-dimensional SOT. In addition, our data support that a two-dimensional sway-referencing paradigm further limits the use of viable proprioceptive cues for postural control, as evidenced by a greater increase in postural sway when compared to performance on the stable support surface conditions. Future studies should investigate the clinical utility of the modified two-dimensional SOT as a means by which to characterize sensory contributions to postural control in older adults, as well as in individuals with balance dysfunction resulting from sensorimotor pathologies, including vestibular hypofunction.

## Data Availability

The raw data supporting the conclusions of this article will be made available by the authors, without undue reservation.

## References

[1] Nashner LM Peters JF. Dynamic posturography in the diagnosis and management of dizziness and balance disorders. Neurol Clin. (1990) 8:331–49. 10.1016/S0733-8619(18)30359-1 2193215

[2] Nashner LM. A model describing vestibular detection of body sway motion. Acta Otolaryngol. (1971) 72:429–36. 10.3109/00016487109122504 5316344

[3] Nashner LM Black FO Wall C. Adaptation to altered support and visual conditions during stance: patients with vestibular deficits. J. Neurosci. (1982) 2:536–44. 10.1523/JNEUROSCI.02-05-00536.1982 6978930 PMC6564270

[4] Horak FB Nashner LM Diener HC. Postural strategies associated with somatosensory and vestibular loss. Exp Brain Res. (1990) 82:167–77. 10.1007/BF00230848 2257901

[5] Black FO Peterka RJ Shupert CL Nashner LM. Effects of unilateral loss of vestibular function on the vestibulo-ocular reflex and postural control. Ann Otol Rhinol Laryngol. (1989) 98:884–9. 10.1177/000348948909801109 2817680

[6] Pedalini MEB Cruz OLM Bittar RSM Lorenzi MC Grasel SS. Sensory organization test in elderly patients with and without vestibular dysfunction. Acta Otolaryngol. (2009) 129:962–5. 10.1080/00016480802468930 19437166

[7] Ford-Smith CD Wyman JF Elswick RK Fernandez T Newton RA. Test-retest reliability of the sensory organization test in noninstitutionalized older adults. Arch Phys Med Rehabil. (1995) 76:77–81. 10.1016/s0003-9993(95)80047-6 7811180

[8] Maki BE Holliday PJ Topper AK. A prospective study of postural balance and risk of falling in an ambulatory and independent elderly population. J Gerontol. (1994) 49:M72–84. 10.1093/geronj/49.2.m72 8126355

[9] Kannus P Leiponen P Parkkari J Palvanen M Järvinen M. A sideways fall and hip fracture. Bone. (2006) 39:383–4. 10.1016/j.bone.2006.01.148 16504612

[10] Nilsson M Eriksson J Larsson B Odén A Johansson H Lorentzon M. Fall risk assessment predicts fall-related injury, hip fracture, and head injury in older adults. J Am Geriatr Soc. (2016) 64:2242–50. 10.1111/jgs.14439 27689675

[11] Greenspan SL Myers ER Kiel DP Parker RA Hayes WC Resnick NM. Fall direction, bone mineral density, and function: risk factors for hip fracture in frail nursing home elderly. Am. J. Med. (1998) 104:539–45. 10.1016/s0002-9343(98)00115-6 9674716

[12] Rosiak O Puzio A Kaminska D Zwolinski G Jozefowicz-Korczynska M. Virtual reality-A supplement to posturography or a novel balance assessment tool? Sensors (Basel). (2022) 22:7904. 10.3390/s22207904 36298254 PMC9608655

[13] Pletcher ER Williams VJ Abt JP Morgan PM Parr JJ Wohleber MF Normative data for the NeuroCom sensory organization test in US military special operations forces. J Athl Train. (2017) 52:129–36. 10.4085/1062-6050-52.1.05 28140624 PMC5343525

[14] Nashner LM Peters JF. Dynamic posturography in the diagnosis and management of dizziness and balance disorders. Neurol Clin. (1990) 8(2):331–49. 2193215

[15] Maurer C Peterka RJ. A new interpretation of spontaneous sway measures based on a simple model of human postural control. J Neurophysiol. (2005) 93:189–200. 10.1152/jn.00221.2004 15331614

[16] Prieto TE Myklebust JB Hoffmann RG Lovett EG Myklebust BM. Measures of postural steadiness: differences between healthy young and elderly adults. IEEE Trans Biomed Eng. (1996) 43:956–66. 10.1109/10.532130 9214811

[17] Wagner AR Kobel MJ Merfeld DM. Impact of canal-otolith integration on postural control. Front Integr Neurosci. (2021) 15:773008. 10.3389/fnint.2021.773008 34970126 PMC8713561

[18] Blanca MJ Alarcón R Arnau J Bono R Bendayan R. Non-normal data: is ANOVA still a valid option? Psicothema. (2017) 29:552–7. 10.7334/psicothema2016.383 29048317

[19] Koo TK Li MY. A guideline of selecting and reporting intraclass correlation coefficients for reliability research. J Chiropr Med. (2016) 15:155–63. 10.1016/j.jcm.2016.02.012 27330520 PMC4913118

[20] Carpenter MG Allum JH Honegger F. Vestibular influences on human postural control in combinations of pitch and roll planes reveal differences in spatiotemporal processing. Exp Brain Res. (2001) 140:95–111. 10.1007/s002210100802 11500802

[21] Black FO. What can posturography tell US about vestibular function? Ann N. Y. Acad Sci (2001) 942:446–64. 10.1111/j.1749-6632.2001.tb03765.x 11710483

[22] Di Fabio RP. Sensitivity and specificity of platform posturography for identifying patients with vestibular dysfunction. Phys Ther. (1995) 75:290–305. 10.1093/ptj/75.4.290 7899487

[23] Voorhees RL. Dynamic posturography findings in central nervous system disorders. Otolaryngol Head Neck Surg. (1990) 103:96–101. 10.1177/019459989010300114 2117737

[24] Honaker JA Janky KL Patterson JN Shepard NT. Modified head shake sensory organization test: sensitivity and specificity. Gait Posture. (2016) 49:67–72. 10.1016/j.gaitpost.2016.06.024 27372458 PMC5278762

[25] Wrisley DM Stephens MJ Mosley S Wojnowski A Duffy J Burkard R. Learning effects of repetitive administrations of the sensory organization test in healthy young adults. Arch Phys Med Rehabil. (2007) 88:1049–54. 10.1016/j.apmr.2007.05.003 17678669

[26] Hayes WC Myers ER Morris JN Gerhart TN Yett HS Lipsitz LA. Impact near the hip dominates fracture risk in elderly nursing home residents who fall. Calcif Tissue Int. (1993) 52:192–8. 10.1007/BF00298717 8481831

[27] Nevitt MC Cummings SR. Type of fall and risk of hip and wrist fractures: the study of osteoporotic fractures. The study of osteoporotic fractures research group. J Am Geriatr Soc. (1993) 41:1226–34. 10.1111/j.1532-5415.1993.tb07307.x 8227898

[28] Parkkari J Kannus P Palvanen M Natri A Vainio J Aho H Majority of hip fractures occur as a result of a fall and impact on the greater trochanter of the femur: a prospective controlled hip fracture study with 206 consecutive patients. Calcif Tissue Int. (1999) 65:183–7. 10.1007/s002239900679 10441647

[29] Chvatal SA Torres-Oviedo G Safavynia SA Ting LH. Common muscle synergies for control of center of mass and force in nonstepping and stepping postural behaviors. J Neurophysiol. (2011) 106:999–1015. 10.1152/jn.00549.2010 21653725 PMC3154805

[30] Torres-Oviedo G Macpherson JM Ting LH. Muscle synergy organization is robust across a variety of postural perturbations. J Neurophysiol. (2006) 96:1530–46. 10.1152/jn.00810.2005 16775203

[31] Wagner AR. Uncovering vestibular contributions to age-related imbalance [Internet] [PhD diss.] [Columbus, OH]: The Ohio State University (2023). Available at: http://rave.ohiolink.edu/etdc/view?acc_num=osu1672875969330073

